# Twins, with One Placenta, Two Cords and One Set of Membranes

**Published:** 1866-10

**Authors:** S. A. Ferrin

**Affiliations:** Montfort, Wisconsin


*Twins with One Placenta, Two Cords and One Set of Membranes.* By S. A. FERRIN, of Montfort, Wisconsin.

I was called on July 9th to see Mrs. H——. Found her in bed, lying on her back, entirely unable to help herself. She seemed to be laboring under *general anasarca*, with great dyspnœa and excessive nervous irritability, dread of death, etc. She stated that she was nearly seven months pregnant, and that this bloating had commenced during the third month, and was constantly on the increase. Her bowels were constipated, and she experienced great difficulty in urinating, with constant desire to pass water. I administered, as a gentle laxative, pulv. rhei, ʒi, sodæ bi-carb. ʒi., M.—to be repeated in five hours if the bowels are not moved. Gave also, as an eliminant and diuretic: ℞ Potass. acetat. ʒiv., sp. nit. ether, ʒij., aquæ puræ, ℥ij. M. S. Take a teaspoonful every four hours.

The patient improved under the treatment. The dyspnœa lessened, and the bloating subsided somewhat. I continued the treatment at intervals for nearly three weeks.

August 16th. I was hastily summoned, and found her with all the previous symptoms much aggravated; with dyspnœa, great pain in the back, etc. I made a vaginal examination, and finding the parts much swollen, dry and irritable, with a hard and unyielding cervix, decided to induce premature labor. I ruptured the membranes for that purpose. After twelve hours the pains in the back were much increased; the os was dilated to about the size of a twenty-five cent piece. The pains increasing and the os dilating, I administered fl. ex. ergotæ, ʒi., and in twenty minutes repeated the dose.

The patient dropped into a quiet and easy slumber for forty minutes; after which, violent labor pains set in, and in fifteen minutes more a living child was born. After tying the cord, I attempted to remove the placenta, when, upon following it up, I found the head of another child presenting, which, in ten minutes more, was born, precisely similar in size, sex and features to the first, together with a single placenta, one amnion, one chorion and two cords. The placenta if anything was a little larger than ordinary, having the two cords attached equidistant from its circumference and about three and a half inches apart. Since delivery the anasarca is gradually subsiding, and the mother and her boys are doing well.

